# Development of a Bead-Based Multiplex Genotyping Method for Diagnostic Characterization of HPV Infection

**DOI:** 10.1371/journal.pone.0032259

**Published:** 2012-02-29

**Authors:** Mee Young Chung, Yong-Wan Kim, Su Mi Bae, Eun Hye Kwon, Pankaj Kumar Chaturvedi, Gantumur Battogtokh, Woong Shick Ahn

**Affiliations:** 1 Department of Anesthesiology, College of Medicine, The Catholic University of Korea, Seocho-ku, Seoul, Korea; 2 Cancer Research Institute of Medical Science, The Catholic University of Korea, Seocho-ku, Seoul, Korea; 3 Department of Obstetrics and Gynecology, College of Medicine, The Catholic University of Korea, Seocho-ku, Seoul, Korea; Northeastern University, United States of America

## Abstract

The accurate genotyping of human papillomavirus (HPV) is clinically important because the oncogenic potential of HPV is dependent on specific genotypes. Here, we described the development of a bead-based multiplex HPV genotyping (MPG) method which is able to detect 20 types of HPV (15 high-risk HPV types 16, 18, 31, 33, 35, 39, 45, 51, 52, 53, 56, 58, 59, 66, 68 and 5 low-risk HPV types 6, 11, 40, 55, 70) and evaluated its accuracy with sequencing. A total of 890 clinical samples were studied. Among these samples, 484 were HPV positive and 406 were HPV negative by consensus primer (PGMY09/11) directed PCR. The genotyping of 484 HPV positive samples was carried out by the bead-based MPG method. The accuracy was 93.5% (95% CI, 91.0–96.0), 80.1% (95% CI, 72.3–87.9) for single and multiple infections, respectively, while a complete type mismatch was observed only in one sample. The MPG method indiscriminately detected dysplasia of several cytological grades including 71.8% (95% CI, 61.5–82.3) of ASCUS (atypical squamous cells of undetermined significance) and more specific for high grade lesions. For women with HSIL (high grade squamous intraepithelial lesion) and SCC diagnosis, 32 women showed a PPV (positive predictive value) of 77.3% (95% CI, 64.8–89.8). Among women >40 years of age, 22 women with histological cervical cancer lesions showed a PPV of 88% (95% CI, 75.3–100). Of the highest risk HPV types including HPV-16, 18 and 31 positive women of the same age groups, 34 women with histological cervical cancer lesions showed a PPV of 77.3% (95% CI, 65.0–89.6). Taken together, the bead-based MPG method could successfully detect high-grade lesions and high-risk HPV types with a high degree of accuracy in clinical samples.

## Introduction

Human papillomavirus (HPV) infection is associated with a variety of clinical conditions, ranging from asymptomatic infections to benign and malignant diseases of the genital mucosa [1]. In the only extensively studied host, humans, nearly 100 HPV types have been described based on the isolation of the complete genome [2]. The HPV types have been grouped into high risk and low risk HPV types according to their potential to cause invasive cancer [3]. Studies of the oncogenic potential of HPVs have clearly demonstrated that high risk HPVs are a necessary cause for the development of cervical cancer [4].

There are currently two main molecular approaches used for HPV detection: the polymerase chain reaction (PCR) with generic primers used to amplify part of the L1 gene of the viral capsid, which is highly conserved among anogenital HPVs, and the Hybrid Capture II test (HCII; Digene, Gaithersburg, MD, USA), which detects 13 high risk HPVs including 16, 18, 31, 33, 35, 39, 45, 51, 52, 56, 58, 59 and 68. The HCII allows quantification of the HPV DNA load, but lacks information on the HPV genotype. Since the oncogenic potential differs among the high risk HPV types [5]–[7], viral genotyping, following high risk HPV detection, might be useful for better risk stratification of high risk positive women. The general PCR-based methods followed by PGMY line blot [8] and LiPA line blot [9], targeting the L1 region of the HPV genome, enable the precise identification of the HPV genotypes present in samples. However, these assays are restricted to a maximum of about 40 oligonucleotide probes per hybridization reaction due to the format of the line blot strips and depend on a visual read-out of the signal [10]. In addition, none of these assays can be automated or deployed on a high-throughput platform, features that are essential for an assay intended for use with a large volume of patients.

Luminex (xMAP) suspension array technology is based on polystyrene beads that are internally dyed with various ratios of two spectrally distinct fluorophores. Different molecules such as individual oligonucleotide probes can be coupled to different bead sets with specific absorption spectra. These sets are combined to a suspension array and allow up to 100 different probes to be measured simultaneously in a single reaction (multiplexing). This technology can potentially be fully automated, dramatically decreasing the personal cost component of the assay; this assay has been used for the genotyping of 45 HPV types with PGMY09/11 PCR [11], 22 HPV types with GP5+/6+ PCR [10], 15 HPV types with YBT L1/GP-1 PCR [12], and 18 HPV types with GP5+/6+ PCR [13]. These multiplex HPV genotyping (MPG) methods have been compared with other well established HPV detection methods such as HCII, restriction fragment length polymorphism [14] and DNA chip technology [15] for the evaluation of their performance. However, none of these well established assays are perfect and suitable for the “gold standard”. Sequencing gives the most conclusive genotype information, although it is the most labor intensive [16], [17]. However, sequencing is the most desirable way to validate HPV genotyping methods.

The purpose of the present study was to validate a bead-based MPG method developed for the identification of 20 HPVs (15 high risk HPV types including 16, 18, 31, 33, 35, 39, 45, 51, 52, 53, 56, 58, 59, 66, 68 and 5 low risk HPV types including 6, 11, 40, 55, 70) with sequencing. Histologically confirmed cervical intraepithelial neoplasia (CIN) grade 1 (CIN1), and CIN grades 2–3 or higher (CIN2+) were used. This study showed that the MPG method is a valuable tool for the detection of pre-cervical cancer or cervical cancer lesions (CIN2+).

## Results

**Figure 1:**
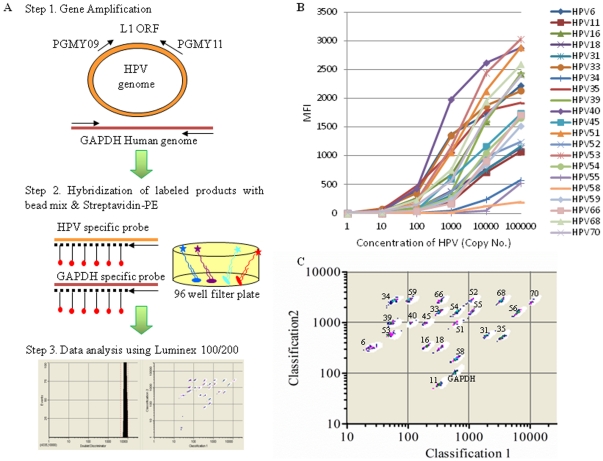
Overall scheme of multiplex bead-based assay and its representative multiplexing results. A) Assay workflow of a bead-based multiplex HPV genotyping (MPG) method showing the general steps required to detect 20 types of HPV. B) Standard curves for the multiplexed HPV genotyping assays in a 96-well microplate format results. The detection limits were determined by using HPV plasmids with serial dilution based on the cutoff value of 150 MFI. C) Dot plot obtained from microspheres encoded with intensities of two fluorochromes. The number is for each type of HPV. The white circles represent each bead type's spectral address; a flow cytometer classifies the bead type and amount of PCR product attached to each bead's surface, expressed as MFI. The sample injection volume was 50 ul, and the system was set to count 100 events for each of the color-coded beads. While the Luminex beads fall within their respective regions for detection and counting, the larger particles fall near the bottom of the graph, and do not interfere with Luminex particle detection and counting. Increasing density is indicated by contrasting colors.

All women underwent cytologic testing and HPV screening. The number of sequencing positive and negative samples was 484 and 406, respectively. Among these, 484 PCR product positive samples were used for the evaluation of the bead-based MPG method as shown in Figure 1A. Among 484 women with HPV infections were 339 controls with negative histology including cervicitis, 32 women with histologically confirmed cervical intraepithelial neoplasia (CIN) grade 1 (CIN1), and 113 women with histologically confirmed CIN grades 2–3 or higher (CIN2+).

### Sensitivity and specificity of the bead-based MPG method

The bead-based MPG method basically uses the MFI of the reporter fluorescence for HPV genotyping. That is, positivity of the respective types is calculated from the MFI over a defined cutoff value and can provide a semi-quantitative numerical output. For the calculation of the cutoff value, the biotinylated PGMY09/11 amplimers generated from 200 ng of the HPV negative human DNA were hybridized after one step PCR (see Materials and Methods for detail). The background value was in the range from 50 MFI to 135 MFI depending on the HPV probes used (Table S1). The detection limits were determined by using HPV plasmids with serial dilution based on the cutoff value of 150 MFI as shown in Figure 1B. The bead-based MPG method successfully detected HPV 16 at 50 plasmid copies while HPV 40 was detected even at five plasmid copies. Less than five thousand plasmids were sufficient for the detection of all 20 HPV types using one step reaction.

To determine the ability of the bead-based MPG method to specifically detect different HPV types, the 20 type-specific probes and the GAPDH probe were coupled individually to defined bead sets (“21-plex”) and hybridized to PMGY09/11 amplicons generated from 1×10^6^ copies of HPV plasmids and 200 ng of genomic DNA from HeLa cells known to be HPV 18 positive. Positivity for the respective types was calculated from the MFI over their cutoff values. The PGMY09/11 PCR amplicons of the HeLa cells showed a high MFI value only in for the HPV 18 probe. There was a 10 fold higher MFI for GAPDH than the other PGMY09/11 PCR amplicons without GAPDH genomic DNA (Table 1). In addition, no significant cross-hybridization signals were generated by the PCR amplicons of individual HPV plasmids. These findings indicate good discrimination with the one step PCR product generated by the biotinylated PGMY09/11 primers.

**Table 1:** Type specificity of the bead-based MPG method.

20 HPV type and HeLa cells		GAPDH probe and HPV type-specific probe																			
	GAPDH	6	11	16	18	31	33	35	39	40	45	51	52	53	55	56	58	59	66	68	70
HeLa	96	10	21	12	6650	15	14	18.5	17	88	15	8	20	22	19	10	14.5	28.5	24	19	22
6	14	5194	73	40	36	18	38	93.5	73	52	40	16	25.5	73	40.5	29	47	105.5	37.5	72	42.5
11	7	9	4079	24	12.5	11.5	17.5	55	33	35	20	9	12.5	37.5	30	16	23	48	18.5	30.5	34.5
16	6	6.5	18	4781	13	12	15	21.5	19	19	13	9	12	16.5	11	9	14	29	12	20	12
18	6	9	16	15.5	5751	10	12	30	24	17	11	6	10.5	18	11	9	13	31	10.5	19	14
31	7.5	10	19.5	20	9.5	2623	13	26	22	18	10	8	12	20	11	10	15	35.5	14	24	13
33	5	7	15	21.5	15	11.5	4903	28.5	21	18	12.5	6.5	11.5	23.5	13	9	15	37	14.5	20	14
35	8	11	27	26.5	15	15	21	6456	34	22.5	17	8.5	13	32	19	13	17	68	24	31	19
39	10	13	27	30.5	12	13	20	44.5	2961	20	21	10	18	37	20.5	12	22.5	53	21	36	22.5
40	8	8	20.5	20	11.5	13	17.5	34.5	28	7172	18	8	15.5	24	19	11	21	45	15.5	26	19
45	6.5	6	18	22	11.5	9	11	21	23	82	3598	7	8	14	13	10	9	33	14	17	11
51	7	8	18	18	13	11	9	27	17.5	20	8	6449	13	19	11	9	12	42.5	15	15	14
52	5	9	14	16	10	10.5	9	22	17	91	12	5	1485	21	10	7	10	32	10	14.5	13
53	5	8	17	21	8	14	11	23	19	85	11	4	13	4631	10	8	11.5	31	12	22	12
55	7	9	19	21	8	10	14	26	27	18	14	8	12	23	2536	10	14	41	12	25	15
56	8	8	13	16	9	9	9	19	16.5	14	9	5	10	13	6	4443	11	26.5	12	18	9
58	3.5	9	24	14	11	13	14	34	39	138	15	8	12	27	14.5	10	2535	48	18.5	30	15
59	5	6	12	12	7	10	8	46	14	11	9.5	7	12	14	8	8	8	5457	11	15.5	8
66	9	8	17	15	10	14	15	22	20	19	9	7	11	17.5	8	9	16	28	2007	20	11
68	3	6	12	12	7.5	10	10	18	17	51	9	5	8	15	7.5	6	9	24	10	5771	9
70	9	16	26	15	14	18	17	39	32	14	17	3	13	26	11	10	12	52	17.5	30	5325

### Analysis of clinical samples and comparison with Sequencing

As shown in Figure 1C, the performance of the bead-based MPG was tested on 484 clinical samples that were PGMY09/11 PCR product positive and the accuracy was verified by sequencing. The bead-based method detected 383 single infections and 101 multiple infections. For the single infections, the 16 HPV genotypes including HPV-6, 11, 18, 31, 35, 40, 45, 56, 59, 66, and 70 showed 100% accuracy while the lowest accuracy (55.6%) was observed for HPV 68 (Table 2). The accuracy for HPV-16, the most common HPV type, was 98.1% even with its high number of samples compared to the other HPV types. The other common single infections were HPV-18, HVP-53 and HPV-58, and the accuracies of these types were 100%, 81.6%, and 87.1%, respectively.

**Table 2:** The accuracy of the bead-based MPG method in single infections.

HPV genotype	No. of positive sampleswith bead-base MPG	No. of samplesconfirmed by sequencing	Accuracy (%)
6	13	13	100.0
11	4	4	100.0
16	105	103	98.1
18	31	31	100.0
31	15	15	100.0
33	7	6	85.7
35	10	10	100.0
39	16	15	93.8
40	2	2	100.0
45	3	3	100.0
51	19	16	84.2
52	25	23	92.0
53	38	31	81.6
55	4	3	75.0
56	20	20	100.0
58	31	27	87.1
59	4	4	100.0
66	9	9	100.0
68	9	5	55.6
70	18	18	100.0
Total number	383	358	93.5

The accuracy of the bead-based MPG method was also estimated for multiple infections (Table S2). The verification of multiple infections was performed by sequencing with the respective type-specific primer. Eighty percent of the total of multiple infections showed a perfect match while a complete type mismatch was observed only in one sample. The double infections containing HPV-16 and 18 accounted for 37.6% of the total number of multiple infections. The double infections containing only low risk HPV types were found in only 5.5% of the samples. The MPG method indiscriminately detected dysplasia of several grade cytology including 71.8% of ASCUS (atypical squamous cells of undetermined significance) and more specific for high grade lesions as shown in Table 3. Of the total women with a positive HPV result and a histological diagnosis (n = 271) including all age groups, 90 women had histological cervical cancer lesions (CIN2+), giving a PPV of 32.3%. For women with HSIL and SCC diagnosis, 32 of 43 women having histological CIN2+ showed a PPV of 74.4%. Among women >40 years of age, the data showed that 22 of 25 women with the same histology showed a PPV of 88%. Of the highest risk HPV types including HPV-16, 18 and 31 positive women of all age groups, 62 of 88 women had a diagnosis of CIN2+, giving a PPV of 70.4% as shown in Table 4. Among women >40 years of age, 34 of 44 women with the same histology showed a PPV of 77.3% (Table 5). This result suggests that with a higher PPV of the test and high-grade histological diagnosis, the MPG method is an important tool in detecting women with the high-grade lesions or high risk HPV types.

**Table 3:** Total women with a positive HPV test and a histological diagnosis.

Cytology	Total with this diagnosis	HPV positives	Women with CIN2+ among the HPV positives	Women with a histology diagnosis	PPV for CIN2+ (95% CI)
Normal	222	94	27	115	23.4 (15.6–31.1)
ASCUS	71	51	15	46	32.6 (19.1–46.1)
LSIL	88	80	16	74	21.6 (12.2–310)
HSIL	32	32	20	29	68.9 (52.1–85.7)
SCC	15	14	12	14	85.7 (67.4–100.0)
Total	428	271	90	278	32.3 (26.8–37.8)

Women >40 years of age					
Normal	90	42	14	59	23.7 (12.8–34.6)
ASCUS	40	28	8	25	32.0 (13.7–50.3)
LSIL	38	36	5	36	13.8 (2.53–25.1)
HSIL	20	20	14	15	93.3 (80.65–100.0)
SCC	10	9	8	10	80.0 (55.2–100.0)
Total	198	135	49	145	33.8 (24.7–39.9)

ASCUS, atypical squamous cells of undetermined significance.

LSIL, low grade squamous intraepithelial lesion.

HSIL, high grade squamous intraepithelial lesion.

SCC, squamous cell cervical carcinoma.

CIN2+, higher cervical intraepithelial neoplasia.

**Table 4:** Total women with a different HPV test and a histological diagnosis.

High riskHPV type	HPV positives	Women with CIN2+ among the HPV positives	Women with a histology diagnosis	PPV for CIN2+(95% CI)
16	105	50	64	78.1 (68.0–88.3)
18	31	9	18	50.0 (26.9–73.1)
31	15	3	6	50.0 (10.0–90.0)
33	7	4	5	80.0 (44.9–100.0)
35	10	1	2	50.0 (0.0–100.0)
39	16	0	9	0
45	3	0	0	-
51	19	1	7	14.3 (0.0–40.2)
52	25	2	13	15.4 (0.0–35.0)
53	38	1	15	6.7 (0.0–19.3)
56	20	0	8	0
58	31	5	11	45.5 (16.0–74.9)
59	4	0	2	0
66	9	1	6	16.7 (0.0–46.5)
68	9	1	4	25.0 (0.0–67.4)
total	342	78	170	45.9 (38.4–53.4)

**Table 5:** Women >40 years of age with a different HPV test and a histological diagnosis.

High riskHPV type	HPV positives	Women with CIN2+ among the HPV positives	Women with a histology diagnosis	PPV for CIN2+(95% CI)
16	36	25	31	80.6 (66.7–94.6)
18	13	6	10	60.0 (29.6–90.4)
31	4	3	3	100
33	4	2	3	66.7 (13.3–100)
35	4	1	2	50.0 (0.0–100)
39	3	0	2	0
45	1	0	0	-
51	8	2	8	25.0 (0.0–55.0)
52	6	1	6	16.7 (0.0–46.5)
53	13	0	13	0
56	4	0	4	0
58	13	5	10	50.0 (19.0–81.0)
59	2	0	2	0
66	5	1	5	20.0 (0.0–55.1)
68	4	1	2	50.0 (0.0–100)
total	120	47	101	46.5 (36.8–56.3)

The HPV genotyping by the bead-based method and sequencing showed almost perfect agreement (κ = 0.935; 95% CI, 0.91–0.96); while the κ values for the detection of HPV 16 and 18 were 0.99 and 0.96, respectively. In addition, the sensitivity, specificity and accuracy of the bead-based MPG method were 90.7%, 100%, and 94.9%, respectively, for the detection of high risk HPVs when the genotype information given by sequencing was used as the standard. This suggests that the bead-based MPG method could successfully detect HPV infections and identify HPV genotypes with a high degree of accuracy.

## Discussion

Accurate HPV genotyping has become an important prognostic indicator in the clinical screening of women due to the strong casual relationship between persistent HPV infection and high grade cervical intraepithelial neoplasia [18], [19]. In cervical screening programs, HPV DNA is typically detected by a non-radioactive signal amplification method, the Hybrid Capture II assay (HC2; Digene Co, Gaithersburg, Md.,USA) which allows for the detection of 13 probable high risk and five low risk HPV types using two different cocktails of RNA probes, in two reactions [20]. HC2 is at present the only Food and Drug Administration-approved assay for routine detection of HPV infections [21]. The HC2 assay provides information on the viral load, which is useful in detecting recurrences after treatment of cervical intraepithelial neoplasia 3 and micro-invasive cancer as well as the response to radiation therapy for cervical cancer [22], [23]. However, the HC2 does not provide information on the specific type of HPV detected. In addition, it is less sensitive than the PCR-based method and the cross-reactivity of the two cocktail probes decreases the clinical specificity of a positive result [20]. The sensitivity and the specificity of HPV DNA can be improved by PCR-based methods including restriction fragment length polymorphism (RFLP) assays, reverse hybridization analysis, microarray platforms and/or sequencing. Among these methods, sequencing is the “gold standard” for HPV genotyping, in which HPV DNA is amplified with primers such as GP5+/6+ (consensus) or MY09/11 (degenerate) followed by sequencing. Since the HPV genotyping by sequencing is the most accurate method, it is desirable to evaluate the accuracy of any HPV genotyping method on the basis of the sequencing results [16], [17]. In this study, the one step PCR product generated by the biotinylated PGMY09/11 primer showed good discrimination for 484 HPV positive samples and evaluated by sequencing. The accuracy of the MPG method was 93.5% (95% CI, 91.0–96.0) and 80.1% (95% CI, 72.3–87.9) for single and multiple infections, respectively. This indicates that the HPV genotype of clinical samples could be successfully identified by the bead-based MPG method with a high degree of accuracy and one step reaction.

In most studies, the HPV genotyping accuracy in multiple infections has not been evaluated because the conventional direct sequencing cannot resolve the identity of genotypes in samples containing multiple infections in which viral sequences overlap [16], [24], [25]. Recent study showed that sequencing was able to identify types in only four cases of multiple infection, which were those having only two virus types in the sample [26]. In the multiple infection, the sequencing yields nonspecific amplification products, which causes nonspecific and uninterruptible sequencing data. Therefore, it was suggested that both sequencing and type-specific PCR could be employed as a genotyping strategy for HPV in clinical practice. In this study, multiple infections were confirmed by repeated sequencing analysis with type-specific primers. The high accuracy of the bead-based MPG method in multiple infections might be due to the efficient hybridization kinetics of Luminex suspension array, which closely approximates the kinetics of solution-phase hybridization [11] and results in short hybridization times and faster turn-around times that is possible with the DNA chip. In addition, the quality of the bead-based MPG method is more controllable than with the DNA chip; this is because its performance can be tested by sampling the master array of beads.

This study also showed that 89.3% (95% CI, 86.2–92.4) of single infections were caused by high risk types; the three main high risk types were HPV-16, 18, and 53. In the contrast to other studies [15], [27], the frequency of HPV-53 was as high as HPV-18. This suggests that HPV-53 might also play an important role in the development of cervical cancer in Korean women. Studies of sequential HPV acquisition have shown that co-infection is common and most women experience multiple infections over time [28], [29]. Multiple infections in the present study accounted for 20.9% (95% CI, 14.2–27.6) of all HPV infections. Ninety five percent of double infections carry at least one high risk type of HPV and 15% of them occurred within the same subgroup, A7, including HPV-16, 31, 33, 35, 52, and 58. HPV-16 was most frequently detected in double infections. This supports the findings that preexisting HPV-16 was generally associated with an increased risk for subsequent acquisition of other HPV genotypes (both phylogenetically related and unrelated) [30]. All of multiple infections contain at least one high risk type while 39% of them carry only high risk types. It was shown that the risk of co-infection was higher for types 6, 11, 16, 18, 31, 33, 51, 52, and 56 where all types except for types 6 and 11 are high risk HPV [31]. This indicates that the high risk type of HPV might play an important role in multiple infections. Multiple infections might carry a lower risk for malignant progression than single infections with high risk types of HPV, due to the suppressive effect of E2 protein by a type of HPV on E6 and E7 ORFs of another type HPV; because a significantly higher proportion of double infections were detected in women with normal or inflamed cervices than among women with cervical intraepithelial neoplasia 1 or higher severity [32]. However, the number of HPV types showed a significant relationship with the PPV of the cervical intraepithelial neoplasia grade. Of the highest risk HPV types including HPV-16, 18 and 31 positive women >40 years of age, 34 of 44 women with histology had a diagnosis of CIN2+, giving a PPV of 77.3% (95% CI, 65.0–89.6). (70.4% for women of all age). In this case, HPV 16 positive women have a PPV for CIN2+ of 80.6% (95% CI, 66.7–94.6), suggesting that HPV 16 positive women can get a better clinical safety by direct treatment.

In this study, such relationships with multiple infections and cervical intraepithelial neoplasia grade were also observed. The PPV for HPV positive women with ASCUS is higher than the PPV for women with normal cytology, indicating that the consistency might be due to the high accuracy of the bead-based genotyping method with enough sample numbers. Among women >40 years of age, the PPV of the diagnoses HSIL and SCC for CIN2+ were 93.3% and 80.0% respectively. This suggests that women >40 years of age with HSIL and a positive HPV test can be directly referred to treatment. HPV 16 positive women have a PPV for CIN2+ of 78.1% when including all age groups, representing a higher PPV than 68.9% of the cytological HSIL diagnosis, while the PPV of the other HPV types is lower. Thus, these findings suggest that the bead-based MPG method showed a higher clinical specificity and positive predictive value, specially for CIN2+. For the fundamental understanding of multiple infections and patient follow up for CIN2+ [33], the specific biological features of individual HPV types such as integration status and RNA expression, might be also needed [34]. In addition, a study on the interaction among different HPV types is necessary for achieving better understanding of multiple infections. HPV-HPV interactions might occur through indirect interactions that result from alterations of the host environment or immunological interactions in addition to the direct interactions of HPV genes or gene products.

The accurate genotyping of human papillomavirus is clinically important because the oncogenic activity of HPV is dependent on the specific genotypes. Although HPV genotyping by sequencing is the most accurate method, it is the most labor intensive and time-consuming. Therefore, the ideal HPV genotyping method should have genotyping accuracy comparable to sequencing and be automated or deployed on a high-throughput platform, features essential for an assay intended for a large volume of patients. Our findings suggest that the bead-base MPG method is a valuable strategy for an efficient HPV genotyping and risk stratification of women with a positive HPV test.

## Materials and Methods

### Ethics statement

All patients involved in the study had signed a declaration of consent stating that the patients specimens may be used for scientific intentions. Specimens were obtained from the patients in the Department of Obstetrics and Gynecology in concordance with procedures approved by the Institutional Review Board of The Catholic University of Korea (07BR238).

### Samples and isolation of DNA

A total of 890 cervical samples were tested for HPV detection with the patient's permission, in the Department of Obstetrics and Gynecology, The Catholic University of Korea. Cervical swabs were obtained with a Cerex-Brush (Pappete®, Wallach Surgical Devices, Inc., Orange, CT.,USA). The brush was immediately rinsed in a vial of PreservCyt® solution (Cytyc Corpor., Boxborough, MA.,USA). To isolate DNA, two millimeters of cell suspension was transferred to a 2-ml reaction tube and washed twice by centrifugation at 14,000 rpm for 2 min, followed by removal of the supernatant and resuspension of the pellet in 1 ml of phosphate-buffered saline. The pellet was resuspended in 200 µl of phosphate-buffered saline and used for DNA isolation with the Qiagen blood DNA minikit (Qiagen, Hilden, Germany) according to the manufacturer's instructions.

### Plasmid clones

For the evaluation of specificity and cross hybridization of the bead-based MPG method, plasmid clones of the following 20 HPV types were used: 6, 11, 16, 18, 31, 33, 35, 39, 40, 45, 51, 52, 53, 55, 56, 58, 59, 66, 68 and 70.

### Oligonucleotide probes for HPV genotyping

The type-specific oligonucleotide probes were designed for 20 different HPVs (15 high risk HPV types including 16, 18, 31, 33, 35, 39, 45, 51, 52, 53, 56, 58, 59, 66, 68 and 5 low risk HPV types including 6, 11, 40, 55, 70) on the basis of the DNA sequences of the viral L1 gene, which were obtained from the HPV database (http://hpv-web.lanl.gov/) (Table S3). All probes had 5′-amine modification for bead coupling as well as 15 bp oligo-dT sequences attached for hybridization flexibility.

### Amplification of the L1 fragment and its labeling

Amplification of the L1 gene on 200 ng of genomic DNA isolated from cervical samples was carried out with the biotinylated PGMY09/11 primer set. PCR conditions were the same as published in the PGMY 09/11 version [8]. For a positive control, of the PCR, two primers were used for amplification of glyceraldehyde-3-phosphate dehydrogenase (GAPDH) sequences: GAPDH F, 5′-GAGTCAACGGATTTGGTCGT-3′ for the forward primer and GAPDH R, 5′-TTGATTTTGGAGGCATCTCG-3′ for the reverse primer. Since the copy number of the GAPDH gene in the clinical sample was usually higher than that of the HPV, the GAPDH primer concentration was reduced to one fourth of the PGMY09/11 primer concentration. A biotinylated primer set was used for one step PCR reaction. The PCR components in 20 µL PCR mixture were 0.025 µM biotinylated PGMY09/11 primer, 40 µM dATP, dGTP, dTTP mixture, 10 µM biotin-11-dCTP (Invitrogen, Carlsbad, CA, USA), 75 mM Tris HCl (pH 9.0), 20 mM MgCl_2_, 50 mM KCl, 20 mM (NH_4_)_2_SO_4_, and 1.5 U *Taq* polymerase (Biotools, Madrid, Spain). A 5-min denaturation step at 94°C was followed by a 40-cycle denaturation step at 94°C for 1 min, an annealing step at 53°C for 1 min and an elongation step at 72°C for 1 min.

### Coupling of oligonucleotide probes to beads

The type-specific oligonucleotide probes were 5′-amine modified and then were coupled to carboxylated beads (Luminex Corp., Austin, TX, USA) through a carbodiimide base coupling procedure. Preparation of the beads coupled with oligonucleotide followed a procedure reported previously [12]. Each bead carrying the type-specific oligonucleotide was finally mixed in equal proportion. Here, this mixture is referred to as the bead mix.

### Hybridization assay

Hybridization buffer (21 µL of 2× EZway Hybrisol; Koma Co., Seoul, Korea) and 1 µL of HPV bead mix containing 6.75×10^4^ beads (2.5×10^3^ for each HPV type and control) were added to a labeling tube containing the PCR product. The hybridization was conducted as previously reported [12]. After hybridization, the samples were transferred to a filter plate immediately and washed three times with washing buffer (0.2 M NaCl, 0.1 M Tris, 0.08% Triton X-100, pH 8.0) at room temperature. The beads were resuspended for 15 min in 100 µL of streptavidin-R-phycoerythrin (Strep-PE; Molecular Probes, Eugene, OR, USA) and analyzed for internal bead color and R-phycoerythrin receptor fluorescence on a Luminex 100 analyzer. The median reporter fluorescence intensity (MFI) of at least 100 beads was computed for each bead set in the sample.

### Sequencing

The amplified L1 gene with the PGMY09/11 primer set was purified with the QIAquick PCR Purification Kit (Qiagen) according to the manufacturer's instructions. Sequencing was performed using the BigDye Terminator v3.1 cycle sequencing kit with AmpliTaq DNA polymerase (Applied Biosystems) according to the protocols supplied by the manufacturer with type-specific primers. The sequences obtained from the sequencing were compared with the HPV sequences of known types using the basic local alignment search tool from the NCBI website (http://www.ncbi.nlm.nih.gov/BLAST).

### Cutoff definition and statistical analysis

Reactions of the probes with the PCR products derived from the PGMY09/11 PCR on 200 ng of HPV negative genomic DNA was considered the background values. In this study, the HPV negative genomic DNA was extracted from the human cervical cancer cell line C33A. The background was calculated by the mean value of 19 replicates and the cut-off value was 150 MFI, which is slightly higher than the highest background of 135 MFI, for all HPV probes. Agreement between the bead-based MPG method and sequencing was assessed by the Cohen κ statistic, with values of 0.00 to 0.20 indicating poor agreement; 0.21 to 0.40, fair agreement; 0.41 to 0.60, moderate agreement; 0.61 to 0.8, substantial agreement; and 0.81 to 1.00 almost perfect agreement.

## Supporting Information

Table S1 **Detection limits of 20 type-specific probes used for the bead-based MPG method.**
(DOC)

Table S2 **The comparison of the results of HPV genotyping with DNA sequencing.**
(DOC)

Table S3 **Type-specific oligonucleotide probes for 20 HPV genotypes.**
(DOC)
