# Supplementary material for: Development of a Bead-Based Multiplex Genotyping Method for Diagnostic Characterization of HPV Infection
Source: PLoS One. 2012 Feb 29;7(2):e32259. doi: 10.1371/journal.pone.0032259 (PMC3290557; doi:10.1371/journal.pone.0032259)
Supplement: Table S1 — Detection limits of 20 type-specific probes used for the bead-based MPG method. (DOC) [file pone.0032259.s001.doc]

Table S1. Detection limits of 20 type-specific probes used for the bead-based MPG method.

| HPV type-specific probe | | | | | | | | | | | | | | | | | | | | | |
| --- | --- | --- | --- | --- | --- | --- | --- | --- | --- | --- | --- | --- | --- | --- | --- | --- | --- | --- | --- | --- | --- |
|  |  | 6 | 11 | 16 | 18 | 31 | 33 | 35 | 39 | 40 | 45 | 51 | 52 | 53 | 55 | 56 | 58 | 59 | 66 | 68 | 70 |
|  | Background | 62* | 77 | 110 | 64 | 81 | 85 | 113 | 90 | 135 | 110 | 124 | 85 | 58 | 94 | 74 | 98 | 86 | 53 | 53 | 84 |
| Plasmid copy number | 5X10^0^ | 4 | 65 | 57 | 39 | 40 | 13 | 57 | 45 | 258 | 51 | 53 | 62 | 58 | 35 | 0 | 39 | 43 | 43 | 9 | 44 |
|  | 5X10^1^ | 13 | 85 | 170 | 70 | 73 | 104 | 127 | 47 | 396 | 48 | 83 | 111 | 39 | 33 | 26 | 51 | 49 | 23 | 13 | 47 |
|  | 5X10^2^ | 360 | 1135 | 821 | 358 | 311 | 563 | 471 | 268 | 3168 | 31 | 357 | 247 | 204 | 98 | 80 | 81 | 138 | 60 | 217 | 90 |
|  | 5X10^3^ | 1654 | 4326 | 4766 | 3266 | 2350 | 2526 | 3695 | 1422 | 13092 | 187 | 1878 | 1009 | 1976 | 1048 | 761 | 335 | 697 | 169 | 1973 | 697 |
|  | 5X10^4^ | 5656 | 6686 | 8872 | 9146 | 5341 | 5341 | 9630 | 2549 | 17611 | 591 | 8620 | 2227 | 3773 | 3274 | 3038 | 1135 | 3841 | 536 | 4339 | 5365 |
|  | 5X10^5^ | 7549 | 7076 | 10817 | 12085 | 5946 | 5338 | 11963 | 3958 | 17384 | 3251 | 10785 | 2421 | 6586 | 9667 | 4635 | 1483 | 4970 | 1316 | 4945 | 7824 |
|  | 5X10^6^ | 8724 | 7716 | 10928 | 11555 | 6000 | 6941 | 11701 | 4203 | 16838 | 6555 | 10729 | 3055 | 6343 | 7793 | 5263 | 2121 | 6229 | 1583 | 5286 | 7545 |

*The MFI values were obtained from PGMY09/11 amplimers generated from different copy number of HPV plasmids.
