# Supplementary material for: Development of a Bead-Based Multiplex Genotyping Method for Diagnostic Characterization of HPV Infection
Source: PLoS One. 2012 Feb 29;7(2):e32259. doi: 10.1371/journal.pone.0032259 (PMC3290557; doi:10.1371/journal.pone.0032259)
Supplement: Table S2 — The comparison of the results of HPV genotyping with DNA sequencing. (DOC) [file pone.0032259.s002.doc]

Table S2. The comparison of the results of HPV genotyping with DNA sequencing.

|  | Single infection | Total number of multiple infected sample | | Negative | Total |
| --- | --- | --- | --- | --- | --- |
|  |  | Double infection | More than Double infection |  |  |
| No. of tested sample by sequencing in Luminex positive samples | 383 | 73 | 28 | 406 | 890 |
| No. of sample obtained by sequencing | 358 | 61 | 20 | 406 | 845 |
| Comparison between the results of Luminex and sequencing | Agreement (358/383=93.5%)  Partial agreement (21/383=5.5%) Disagreement (4/383=1%) | Agreement  (61/73=83.5%)  Partial agreement (11/73=15.1%) Disagreement  (1/73=1.4%) | Agreement  (20/28=71.4%)  Partial agreement (8/28=28.6%) | Agreement (406/406=100%) | Agreement  (857/890=96.3%)  Partial agreement (30/890=3.4%)  Disagreement  (3/890=0.3%) |
