# Supplementary material for: Development of a Bead-Based Multiplex Genotyping Method for Diagnostic Characterization of HPV Infection
Source: PLoS One. 2012 Feb 29;7(2):e32259. doi: 10.1371/journal.pone.0032259 (PMC3290557; doi:10.1371/journal.pone.0032259)
Supplement: Table S3 — Type-specific oligonucleotide probes for 20 HPV genotypes. (DOC) [file pone.0032259.s003.doc]

Table S3. Type-specific oligonucleotide probes for 20 HPV genotypes.

| HPV type | GenBank accession no. | Probe sequence (5´-3´) |
| --- | --- | --- |
| 6 | X00203 | ATC CGT AAC TAC ATC TTC CAC ATA CAC CAA |
| 11 | M14119 | ATC TGT GTC TAA ATC TGC TAC ATA CAC TAA |
| 16 | K02718 | GTC ATT ATG TGC TGC CAT ATC TAC TTC AGA AAC |
| 18 | X05015 | TGC TTC TAC ACA GTC TCC TGT A |
| 31 | J04353 | TGT TTG TGC TGC AAT TGC AAA CAG TGA TAC |
| 33 | M12732 | TGC ACA CAA GTA ACT AGT GAC AG |
| 35 | M74117 | GTG CTG CTG TGT CTT CTA GTG A |
| 39 | M62849 | TCT ACC TCT ATA GAG TCT TCC ATA CCT TCT |
| 40 | X74478 | TGC CAC ACA GTC CCC CAC ACC AA |
| 45 | X74479 | ACA CAA AAT CCT GTG CCA AGT A |
| 51 | M62877 | CAC TGC TGC GGT TTC CCC AA |
| 52 | X74481 | GCT GAG GTT AAA AAG GAA AGC A |
| 53 | DQ486475 | TTT CTG CAA CCA CAC AGT CTA TGT CCA |
| 55 | U31791 | TGC TGC TAC AAC TCA GTC TCC ATC TAC A |
| 56 | X74483 | ACT GCT ACA GAA CAG TAA GTA AA T ATG AT |
| 58 | D90400 | TAT GCA CTG AAG TAA CTA AGG AAG GT |
| 59 | X77858 | CTA CTT CTT CTA TTC CTA ATG TAT ACA CAC |
| 66 | U31794 | TGC AGC TAA AAGC ACA TTA ACT AA |
| 68 | AJ831568 | CTG AAT CAG CTG TAC CAA ATA TTT AT |
| 70 | U22461 | TG CAC CGA AAC GGC CAT ACC T |
